# Inorganic Polyphosphate Is Essential for *Salmonella* Typhimurium Virulence and Survival in *Dictyostelium discoideum*

**DOI:** 10.3389/fcimb.2018.00008

**Published:** 2018-01-30

**Authors:** Macarena A. Varas, Sebastián Riquelme-Barrios, Camila Valenzuela, Andrés E. Marcoleta, Camilo Berríos-Pastén, Carlos A. Santiviago, Francisco P. Chávez

**Affiliations:** ^1^Laboratorio de Microbiología de Sistemas, Departamento de Biología, Facultad de Ciencias, Universidad de Chile, Santiago, Chile; ^2^Laboratorio de Microbiología, Departamento de Bioquímica y Biología Molecular, Facultad de Ciencias Químicas y Farmacéuticas, Universidad de Chile, Santiago, Chile; ^3^Laboratorio de Biología Estructural y Molecular, Departamento de Biología, Facultad de Ciencias, Universidad de Chile, Santiago, Chile

**Keywords:** *Salmonella*, *Dictyostelium*, polyphosphate, *ppk*, virulence, intracellular survival, proteomics

## Abstract

Inorganic polyphosphate (polyP) deficiency in enteric bacterial pathogens reduces their ability to invade and establish systemic infections in different hosts. For instance, inactivation of the polyP kinase gene (*ppk*) encoding the enzyme responsible for polyP biosynthesis reduces invasiveness and intracellular survival of *Salmonella enterica* serovar Typhimurium (*S*. Typhimurium) in epithelial cells and macrophages *in vitro*. In addition, the virulence *in vivo* of a *S*. Typhimurium Δ*ppk* mutant is significantly reduced in a murine infection model. In spite of these observations, the role played by polyP during the *Salmonella*-host interaction is not well understood. The social amoeba *Dictyostelium discoideum* has proven to be a useful model for studying relevant aspects of the host-pathogen interaction. In fact, many intracellular pathogens can survive within *D. discoideum* cells using molecular mechanisms also required to survive within macrophages. Recently, we established that *S*. Typhimurium is able to survive intracellularly in *D. discoideum* and identified relevant genes linked to virulence that are crucial for this process. The aim of this study was to determine the effect of a polyP deficiency in *S*. Typhimurium during its interaction with *D. discoideum*. To do this, we evaluated the intracellular survival of wild-type and Δ*ppk* strains of *S*. Typhimurium in *D. discoideum* and the ability of these strains to delay the social development of the amoeba. In contrast to the wild-type strain, the Δ*ppk* mutant was unable to survive intracellularly in *D. discoideum* and enabled the social development of the amoeba. Both phenotypes were complemented using a plasmid carrying a copy of the *ppk* gene. Next, we simultaneously evaluated the proteomic response of both *S*. Typhimurium and *D. discoideum* during host-pathogen interaction via global proteomic profiling. The analysis of our results allowed the identification of novel molecular signatures that give insight into *Salmonella*-*Dictyostelium* interaction. Altogether, our results indicate that inorganic polyP is essential for *S*. Typhimurium virulence and survival in *D. discoideum*. In addition, we have validated the use of global proteomic analyses to simultaneously evaluate the host-pathogen interaction of *S*. Typhimurium and *D. discoideum*. Furthermore, our infection assays using these organisms can be exploited to screen for novel anti-virulence molecules targeting inorganic polyP biosynthesis.

## Introduction

The ability of *Dictyostelium discoideum* cells to feed on bacteria has prompted the development of virulence assays for identifying host defense mechanisms and deciphering bacterial virulence factors (Cosson et al., 2002; Froquet et al., 2009). Basic cellular processes such as phagocytosis, phagosomal development and autophagy, are evolutionarily well conserved between *Dictyostelium* and macrophages (Hägele et al., 2000; Bozzaro and Eichinger, 2011; Dunn et al., 2018). Consequently, *D. discoideum* has been established as a model to study host-pathogen interaction in a wide range of pathogenic bacteria such as *Legionella, Salmonella, Francisella, Mycobacterium*, and *Pseudomonas*, among others (Pukatzki et al., 2002; Hagedorn and Soldati, 2007; Weber et al., 2014; Lampe et al., 2015; Bravo-Toncio et al., 2016; Riquelme et al., 2016; Cardenal-Muñoz et al., 2017). Unlike mammalian phagocytes, *D. discoideum* is amenable to a diverse array of genetic manipulations facilitating the *in vivo* identification of host susceptibility determinants and pathogen virulence factors (Carilla-Latorre et al., 2008; Hasselbring et al., 2011; Pan et al., 2011; Tosetti et al., 2014; Zhang et al., 2016). However, *in vivo* host-pathogen interaction during bacterial infection in *D. discoideum* remains poorly understood.

*Salmonella enterica* serovar Typhimurium (*S*. Typhimurium) is a foodborne pathogen causative of gastroenteritis in a variety of warm-blooded animals that largely relies on its ability to survive inside host cells. Relevant genes required for this process are located in pathogenicity islands such as SPI-1 and SPI-2, which encode two independent type III secretion systems (T3SS-1 and T3SS-2, respectively) that inject effector proteins into host cells and are critical during different stages of infection (reviewed in Haraga et al., 2008). In a previous study, we showed that *S*. Typhimurium genes linked to virulence are required to survive in *D. discoideum*, including those encoding factors involved in the biosynthesis of aromatic compounds, the production of a lipopolysaccharide containing a complete O-antigen, T3SS-1, T3SS-2, the type VI secretion system (T6SS) encoded in SPI-6 and the PhoP/PhoQ two-component system (Riquelme et al., 2016). Hence, *S*. Typhimurium exploits a common set of molecular mechanisms to survive within amoeba and animal host cells, supporting the use of *D. discoideum* as a model for host-pathogen interactions and to study the cellular processes that are affected during infection.

We are particularly interested in inorganic polyphosphate (polyP) metabolism because this biopolymer is important for *D. discoideum* development and predation, and for virulence in many bacterial pathogens (Zhang et al., 2005; Brown and Kornberg, 2008). In fact, we have demonstrated that polyP biosynthesis is essential for *P. aeruginosa* PAO1 virulence toward this amoeba (Bravo-Toncio et al., 2016). Inorganic polyP is an abundant and ubiquitous biopolymer that has been conserved in every cell in nature. In the last decades, an increasing number of physiological functions have been reported for polyP in bacteria (Brown and Kornberg, 2008). Due to their phosphoanhydride bonds similar to those in ATP and their properties as polyanions, polyP serve as microbial phosphagen in a variety of biochemical reactions, as a buffer against alkalis, and as a metal storage and metal-chelating agent. In addition, recent studies have revealed the importance of polyP metabolism in signaling and regulatory processes, cell viability and proliferation, and as modulator of microbial stress response (Gray and Jakob, 2015). In numerous pathogenic bacteria, inactivation of the polyP kinase gene (*ppk*) encoding the enzyme responsible for polyP biosynthesis causes defects in biofilm formation, quorum sensing, motility, general stress and stringent responses, and production of virulence factors (Rao et al., 1998; Rashid and Kornberg, 2000; Rashid et al., 2000a,b; Brown and Kornberg, 2008; Varela et al., 2010; Varas et al., 2017). In *S*. Typhimurium, inorganic polyP is essential for long-term survival and virulence factors production (Kim et al., 2002). However, the exact mechanism that links polyP metabolism and *Salmonella* virulence remains to be elucidated.

In this study, we used *D. discoideum* as a host model to study the link between polyP biosynthesis and virulence in *S*. Typhimurium. To this end, we assessed the intracellular survival of *S*. Typhimurium wild-type and Δ*ppk* strains in the amoeba, and the effect of these strains in the social development of the host. Our results indicate that inorganic polyP is essential during *S*. Typhimurium infection of *D. discoideum*. Also, we used global proteomic profiling to get a global view of host cellular responses toward infection that gave insight into *Salmonella*-*Dictyostelium* interaction.

## Materials and methods

### Bacterial strains and culture conditions

The bacterial strains used in this study are listed in Table 1. All *S*. Typhimurium strains are derivatives of the wild-type, virulent strain 14028s (Fields et al., 1986). Bacteria were routinely grown at 37°C with agitation in Luria-Bertani (LB) medium (10 g/L tryptone, 5 g/L yeast extract, 5 g/L NaCl). When required, LB medium was supplemented with ampicillin (Amp, 100 mg/L), chloramphenicol (Cam, 20 mg/L) or kanamycin (Kan, 75 mg/L). LB medium was solidified by the addition of agar (15 g/L). All procedures involving the use of pathogenic organisms were conducted following the guidelines in the Biosafety Manual of the National Commission of Scientific and Technological Research (CONICYT), and were approved by the Institutional Biosafety Committee of Universidad de Chile, Campus Norte.

**Table 1:** Bacteria and *Dictyostelium* strains used in this study.

**Strains**	**Features**	**Source or reference**
***Salmonella*** **Typhimurium**		
14028s	Wild-type, virulent strain	Laboratory collection
Δ*ppk*	14028s Δ*ppk*::Cam	This study
Δ*ppk*/pPPK	14028s Δ*ppk*::Cam/pPPK	This study
Δ*aroA*	14028s Δ*aroA*::Kan	This study
***Escherichia coli***		
B/r (DBS0348878)	Wild-type strain	Dicty Stock Center (dictyBase)
***Dictyostelium discoideum***		
AX4 (DBS0302402)	*axeA1 axeB1 axeC1*	Dicty Stock Center (dictyBase)

### Construction of mutant strains

All *S*. Typhimurium mutants were generated by the Lambda Red recombination method (Datsenko and Wanner, 2000) with modifications (Santiviago et al., 2009), using plasmid pCLF4 (Kan^R^, GenBank accession number EU629214) or pCLF2 (Cam^R^, GenBank accession number HM047089) as template. Correct allelic replacement in these mutants was confirmed by PCR amplification using primers flanking the substitution site. All primers for PCR amplifications are listed in Table 2.

**Table 2:** Primers used in this study.

**Name**	**Sequence**
aroA_H1+P1	GTTGAGTTTCATGGAATCCCTGACGTTACAACCCATCGCGGTGCAGGCTGGAGCTGCTTC
aroA_H2+P2	AACAGAAGACTTAGGCAGGCGTACTCATTCGCGCCAGTTGCATATGAATATCCTCCTTAG
aroA_Out5	GCGCGCCTCTATCTATAACG
aroA_Out3	TTTTTCATACTAATCTTCCGTTGA
ppk_(H1+P1)	ATGGGTCAGGAAAAGCTATATATCGAGAAAGAACTGAGCTGTGTAGGCTGGAGCTGCTTC
ppk_(H2+P2)	TTAGTCTGGTTGCTCGAGTGATTTGATGTAGTCATAAATTCATATGAATATCCTCCTTAG
ppk_Out5	ACAGGACTGCGTCTGCTTGCCG
ppk_Out3	CTGCATTGCGCCGTCAACCACG
pBAD_Forward	ATGCCATAGCATTTTTATCC
pBAD_Reverse	GATTTAATCTGTATCAGG

*Underlined sequences correspond to the region that anneals to the 5′ or 3′ end of the antibiotic-resistance cassette in template vectors pCLF2 and pCLF4*.

### Construction of complementing plasmid pPPK

A DNA fragment containing the *ppk* gene (including its promoter region) was amplified from the genome of *S*. Typhimurium strain 14028s using *Taq* DNA polymerase (Invitrogen) and primers ppk_Out5 and ppk_Out3 (Table 2). The PCR product was purified from 1% agarose gels using the “QIAquick Gel Extraction Kit” (QIAGEN) and cloned into pBAD-TOPO using the “pBAD-TOPO TA Expression Kit” (Invitrogen). The presence and orientation of the insert in the recombinant plasmid generated (pPPK) was confirmed by PCR amplification using combinations of primers ppk_Out5, ppk_Out3, pBAD_Forward and pBAD_Reverse (Table 2). Finally, *S*. Typhimurium Δ*ppk* was transformed by electroporation with plasmid pPPK for complementation assays.

### *Dictyostelium* culture conditions

*D. discoideum* strain AX4 was obtained from Dicty Stock Center (Kreppel et al., 2004; Basu et al., 2013; Fey et al., 2013), and cultured according to standard protocols (Fey et al., 2007). Briefly, amoebae were maintained at 22°C in SM medium (10 g/L glucose, 10 g/L peptone, 1 g/L yeast extract, 1 g/L MgSO_4_ × 7H_2_O, 1.9 g/L KH_2_PO_4_, 0.6 g/L K_2_HPO_4_, 20 g/L agar), growing on a confluent lawn of *Escherichia coli* B/r. Before development and intracellular survival assays, amoebae were grown in the absence of bacteria (axenic cultures) at 22°C with agitation (180 rpm) in liquid HL5 medium (14 g/L tryptone, 7 g/L yeast extract, 0.35 g/L Na_2_HPO_4_, 1.2 g/L KH_2_PO_4_, 14 g/L glucose, pH 6.3). When required, media were supplemented with streptomycin (Stp; 300 mg/L) and Amp (100 mg/L). Amoebae were harvested in early exponential phase (1–1.5 × 10^6^ cells/mL) and centrifuged at 500 × *g* for 10 min at 4°C. The supernatant was discarded and the pellet was adjusted to 1 × 10^6^ cells/mL in HL5 medium for development assays or washed three times using Soerensen buffer (2 g/L KH_2_PO_4_, 0.36 g/L Na_2_HPO_4_ × 2H_2_O, pH 6.0) for intracellular survival assays. The population of viable amoebae was evaluated by Trypan blue exclusion and counting in a Neubauer chamber.

### Development assay

Individual wells of a 24-well plate containing N agar (Soerensen buffer supplemented with 1 g/L peptone, 1 g/L glucose and 20 g/L agar) were inoculated with 30 μL of a stationary-phase culture from each bacterial strain to be evaluated. The plate was incubated overnight at 22°C to generate bacterial lawns. The next day, 10 μL of a cellular suspension containing 1 × 10^4^ *D. discoideum* AX4 cells in HL5 was spotted in the middle of each well and the plate was further incubated at 22°C for 6 days. Amoebae were monitored daily and the developmental phase reached (“aggregation,” “elevation,” and “culmination”) was scored. A score of “1” was assigned when amoebae aggregated forming a phagocytosis plaque. A score of “2” was assigned when elevated structures, such as “slugs” or “fingers”, were observed all across the surface of the agar in the well. A score of “3” was assigned when fruiting bodies were formed all across the surface of the agar in the well. Intermediate states among two developmental phases were recorded as average values of the corresponding scores. In addition, representative images of *D. discoideum* development were obtained at days 2 and 4 using an Olympus MVX10 stereomicroscope.

### Intracellular survival assay

Intracellular survival assays were performed as described previously (Riquelme et al., 2016). Briefly, each bacterial strain to be assessed was grown to stationary phase, harvested and washed twice with Soerensen buffer. Next, ~2 × 10^7^ *D. discoideum* AX4 cells were mixed with each bacterial strain until reaching a multiplicity of infection (MOI) of 100 bacteria/amoeba in 10 mL of Soerensen buffer. After 1 h of co-incubation at 22°C with agitation (180 rpm), the extracellular bacteria were removed by three sequential washing steps using Soerensen buffer. The infected amoebae were suspended in 10 mL of Soerensen buffer (*t* = 0) and further incubated at 22°C with agitation (180 rpm). Aliquots were obtained at 0, 1, 3, 4.5, and 6 h post infection. The population of viable amoebae were determined at each time point. In parallel, infected amoebae recovered at each time point were lysed with 0.2% Triton X-100 and loads of intracellular bacteria were estimated by serial dilutions and plating on LB agar. Statistical significance was determined using a one-way ANOVA and two-way ANOVA with Fisher's LSD post-test.

### Global proteomic profiling using Q-exactive mass spectrometry

For proteomic analyses, ~1 × 10^6^ amoeba cells were obtained from individual intracellular survival assays after 6 h of infection with the wild-type strain or the Δ*ppk* mutant. Uninfected amoebae were used as control condition. Amoebae from each experimental condition were concentrated by centrifugation at 500 × *g* for 10 min, quick-frozen, and kept at −80°C until further use. Global proteomic profiles from samples representing the different experimental conditions were obtained from Bioproximity, LLC (USA). In each case, a unique proteomic analysis was performed using a pool of cells from three independent assays. Protein denaturation, digestion and desalting of samples were prepared using the filter-assisted sample preparation (FASP) method (Wiśniewski et al., 2009). Briefly, the samples were digested using trypsin, and each digestion mixture was analyzed by ultra-high pressure liquid chromatography (UHPLC-MS/MS) coupled to a high resolution, high mass accuracy quadrupole-Orbitrap mass spectrometer (Q-Exactive, Thermo Fisher). For protein quantification, intensity measurements derived from the area-under-the-curve of the MS/MS scan for each peptide ion identification were summed for each sample. These values were averaged across all samples and a normalization factor determined and applied for each sample (Zhang et al., 2010). MS/MS data were compared with the most recent protein sequence libraries available from UniProtKB. Proteins were required to have one or more unique peptides detected across the analyzed samples with an *e*-value ≤ 0.0001.

Proteomes from each experimental condition were compared using an online tool that generates Venn diagrams and lists of proteins detected in any given condition (http://bioinformatics.psb.ugent.be/webtools/Venn/). The proteins detected in 2 experimental conditions were analyzed by calculating log_2_ values of condition_1/condition_2 detection ratios. Enrichment for a protein in a particular condition was considered when the corresponding calculated value was >0.6 (~1.5 fold enrichment).

### Analysis of *D. discoideum* proteins detected by global proteomic profiling

*D. discoideum* proteins were identified from Q-proteomics data using library ID: 44689 (PubMed Taxonomy database), which include proteomes of strains AX2, AX3 y AX4. Proteins were classified according to the predicted functions annotated in the *Clusters of Orthologous Groups of Proteins* (COGs) database (Tatusov et al., 2000). The UniProtKB ID of each protein was mapped to the COGs database of the Social Amoeba Comparative Genome Browser (SACGB, http://sacgb.leibniz-fli.de/cgi/cog.pl?ssi=free), and the EggNOG database of orthologous groups and functional annotation (Jensen et al., 2008). To do this, both databases were downloaded, and mappings were performed using custom Python scripts. Using this approach we were able to assign COG categories for ~80% of all proteins detected in each experimental condition.

In addition, overrepresentation analyses were performed using the *Protein Annotation Through Evolutionary Relationship* (PANTHER) tool (http://www.pantherdb.org/), a comprehensive system that combines gene function, ontology, pathways and statistical analysis tools that enable the analysis of large-scale, genome-wide data from sequencing, proteomics or gene expression experiments (Mi et al., 2013, 2016). To do this, the UniProtKB IDs from the different sets of detected protein were used as input in PANTHER. This tool was able to map the UniProtKB ID for ~96% of all proteins detected in each experimental condition. The results of overrepresentation analyses were filtered according to three annotation data sets: “Biological process”, “Cellular component”, and “Reactome pathways”. The cut-off for the analyses was set to *P* < 0.05.

### Analysis of bacterial proteins detected by global proteomic profiling

*S*. Typhimurium proteins were identified from Q-proteomics data using the libraries ID: 588858 and ID: 99287 (PubMed Taxonomy database), which include proteomes of strains 14028s and LT2. Virulence-related proteins were assigned following inspections of databases PATRIC_VF, VFDB and VICTORS, using tools available at the Pathosystems Resource Integration Center web site (PATRIC; www.patricbrc.org). Also, proteins encoded in genes located in close proximity to known virulence determinants, as well as genes located within *Salmonella* pathogenicity islands (SPIs) or prophages, were identified and analyzed using Artemis V.14, IslandViewer 3, Pathogenicity Island DataBase (PAIDB v2.0), PHAST and Islander (Rutherford et al., 2000; Zhou et al., 2011; Dhillon et al., 2015; Hudson et al., 2015; Yoon et al., 2015).

## Results and discussion

### Inorganic polyphosphate is essential for *S*. Typhimurium virulence in *D. discoideum*

To evaluate the role played by polyP biosynthesis in the virulence of *S*. Typhimurium in *D. discoideum*, we constructed a Δ*ppk* derivative of the wild-type, virulent strain 14028s. This mutant is impaired in the synthesis of polyP. In addition, the mutant was transformed with a plasmid harboring a wild-type version of *ppk* (i.e., pPPK) to confirm the specificity of the effects attributed to the inactivation of this gene in our assays.

Previous reports indicate that virulent pathogenic bacteria delay the social development of *D. discoideum*, while attenuated or non-pathogenic bacteria allow its rapid progression (within 3–4 days in our experimental conditions). Thus, assessing the effect of a given bacterial strain on the social development of *D. discoideum* can be used to evaluate its virulence (Sillo et al., 2011; Bravo-Toncio et al., 2016; Ouertatani-Sakouhi et al., 2017). Notably, this host-pathogen interaction model has been recently used to identify compounds that inhibit bacterial virulence (Bravo-Toncio et al., 2016; Ouertatani-Sakouhi et al., 2017). Therefore, we compared the effect of feeding *D. discoideum* with the wild-type strain or its Δ*ppk* derivative on the social development of the amoeba. In addition, a Δ*aroA* derivative of strain 14028s (known to be attenuated in murine infection models Sebkova et al., 2008), and *Escherichia coli* B/r (routinely used to feed *D. discoideum* during growth under laboratory conditions Fey et al., 2007) were included in our assay as attenuated and non-pathogenic controls, respectively.

**Figure 1:**
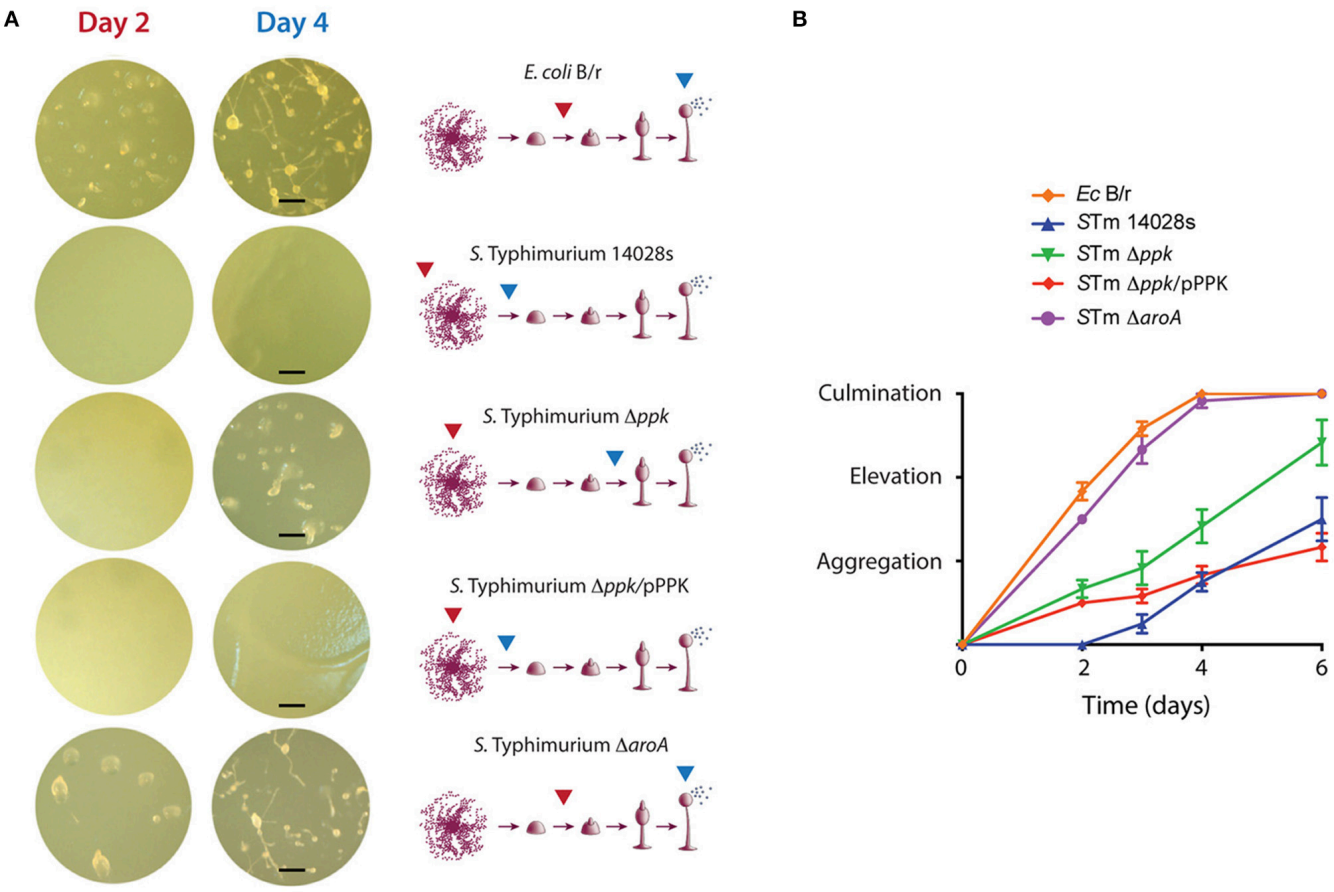
Development of *D. discoideum* co-incubated with *S*. Typhimurium 14028s derivatives and *E. coli* B/r. **(A)** Representative pictures of amoebae development in presence of each bacterial strain. The development stages reached at days 2 and 4 are indicated with red and blue arrows, respectively. Scale bars, 100 μm. **(B)** Development progress evaluated using a numerical scale defined according to the developmental phase reached at each time point (see Materials and Methods section). Graph shows mean values ± SD from 10 independent assays.

The different bacterial strains were inoculated on N agar and incubated overnight to generate bacterial lawns. Then, *D. discoideum* cells were deposited on top of each bacterial lawn and the plates were monitored for 6 days to follow the progression of *D. discoideum* development, which mainly involves three sequential stages: aggregation, elevation, and culmination (Figure 1). *E. coli* B/r allowed the rapid progression of the development, culminating within 3 to 4 days, where mature fruiting bodies were predominant (Figure 1). A similar phenotype was observed in the case of the Δ*aroA* mutant, indicating that this strain is not virulent for *D. discoideum*. This observation is in agreement with previous reports indicating that an *aroA* mutant of *S*. Typhimurium is unable to survive intracellularly in this amoeba (Riquelme et al., 2016). On the contrary, the wild-type strain produced a delay in the development of the amoeba where only the aggregation phase was reached after 6 days of co-incubation (Figure 1). This phenotype was not observed in the case of the Δ*ppk* mutant, which allowed the development of the amoebae until reaching the elevation phase at 6 days of co-incubation. Of note, the Δ*ppk* mutant harboring plasmid pPPK showed a wild-type phenotype (Figure 1). These results indicate that polyP synthesis is essential for *S*. Typhimurium virulence in *D. discoideum*.

Our observations on the interaction of *S*. Typhimurium with *D. discoideum* are consistent with a previous study that evaluated the virulence of the pathogen by assessing its effect on the social development of the amoeba. The authors reported that *Dictyostelium* growth on a lawn of wild-type *S*. Typhimurium causes the aberrant development of the amoeba, or even resulted in cell death depending on nutrient conditions of the medium used for the assay. Both phenotypes required a functional T3SS-2 (Sillo et al., 2011). Additionally, the effect observed for a polyP deficiency on the social development of the amoeba has been also reported in the case of *P. aeruginosa* (Zhang et al., 2005; Bravo-Toncio et al., 2016) highlighting the essential role played by polyP biosynthesis in bacterial virulence using this model organism.

### Inorganic polyphosphate is essential for *S*. Typhimurium survival in *D. discoideum*

Recently, we reported that wild-type *S*. Typhimurium can survive within *D. discoideum* and requires relevant genes linked to virulence for this process (Riquelme et al., 2016). To evaluate the role played by polyP in the intracellular survival of *S*. Typhimurium in *D. discoideum*, we performed infection assays where vegetative amoebae were co-incubated with the wild-type strain or its Δ*ppk* derivative. At different times post infection, intracellular bacteria were recovered from infected amoebae and titrated. The attenuated Δ*aroA* mutant was included as a control in our assays.

**Figure 2:**
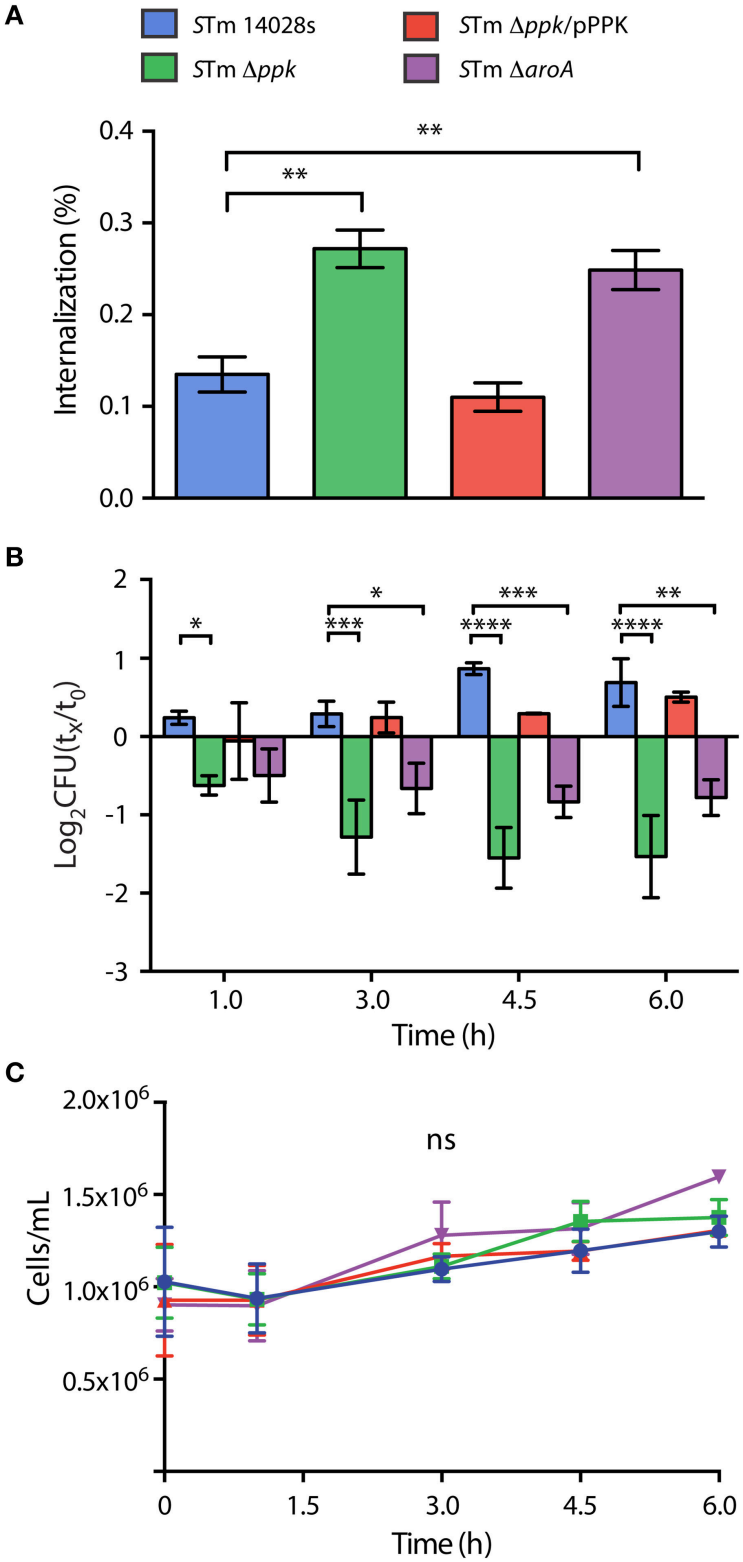
Internalization and intracellular survival of *S*. Typhimurium 14028s derivatives in *D. discoideum*. **(A)** Percentage of internalized bacteria after 1 hour of co-incubation, calculated as 100 × (CFU_t=0_/CFU_inoculum_). Statistical significance was determined using a one-way ANOVA with Fisher's LSD post-test (^**^ = *p* <0.005). **(B)** Intracellular survival at different times post infection, expressed as log_2_(CFU_t=x_/CFU_t=0_). Statistical significance was determined using a two-way ANOVA with Fisher's LSD post-test (^*^ = *p* < 0.05, ^**^ = *p* < 0.005, ^***^ = *p* < 0.001, ^****^ = *p* <0.0001). **(C)** Population of viable amoebae at each time point expressed as cells/mL. Statistical significance was determined using a two-way ANOVA with Fisher's LSD post-test (ns = not significant). Graphs in panels **(A–C)** show mean values ± SD from 5 independent assays. The color code in panel **(A)** is also valid for panels **(B,C)**.

First, we evaluated the internalization of each mutant strain after 1 h of co-incubation with the amoebae, and observed that Δ*ppk* and Δ*aroA* mutants were internalized at higher levels than the wild-type strain. In contrast, the Δ*ppk* mutant harboring plasmid pPPK was internalized at wild-type levels (Figure 2A). Then, we evaluated the intracellular survival of each strain at different times post infection and observed that the wild-type strain was able to survive and replicate in the amoebae. On the contrary, the Δ*ppk* mutant was defective for intracellular survival at all time points evaluated. The same phenotype was observed in the case of the attenuated Δ*aroA* mutant. The intracellular survival of the Δ*ppk* mutant harboring plasmid pPPK was comparable to that shown by the wild-type strain (Figure 2B). It is worth mentioning that no effect in amoeba viability was observed during the course of these experiments (Figure 2C), indicating that the differences observed in the titers of intracellular bacteria are not attributable to changes in the number of viable amoebae.

Overall, our results indicate that polyP synthesis is essential for *S*. Typhimurium to survive intracellularly in *D. discoideum*. These observations are in line with a previous study indicating that a Δ*ppk* mutant of *S*. Typhimurium is deficient for intracellular survival in RAW 264.7 murine macrophages (Kim et al., 2002). In addition, several studied indicate that *S*. Typhimurium *ppk* mutants present a variety of phenotypes, including defective long-term survival *in vitro*, defective responses to oxidative stress and starvation, sensitivity to polymyxin, intolerance to acid and heat, impaired invasiveness in HEp-2 epithelial cells, and loss of swimming motility, all of which strongly influence virulence (Kim et al., 2002; McMeechan et al., 2007; Cheng and Sun, 2009). Accordingly, it has been reported that a *ppk* mutant of *S*. Typhimurium was attenuated in orally-infected Rhode Island Red chickens and BALB/c mice (McMeechan et al., 2007).

### Global proteomic profiling of *Dictyostelium-salmonella* interaction

In order to determine the global response of *D. discoideum* to infections with *S*. Typhimurium wild type or its Δ*ppk* mutant derivative, we performed a global proteomic profiling of such interactions. To achieve this, the amoebae were co-incubated with each bacterial strain until reaching 6 h of infection. This time was chosen because the intracellular survival of both strains in *D. discoideum* showed the highest differences (Figure 2B). Non-infected amoebae were used as control condition. Next, shotgun proteomic profiling of infected and control amoebae were performed by UHPLC-MS/MS (Q-proteomics). Thus, 1779, 1950, and 1850 proteins were detected in samples of amoebae infected with the wild-type strain, amoebae infected with the Δ*ppk* mutant, and uninfected amoebae, respectively (Table S1).

**Figure 3:**
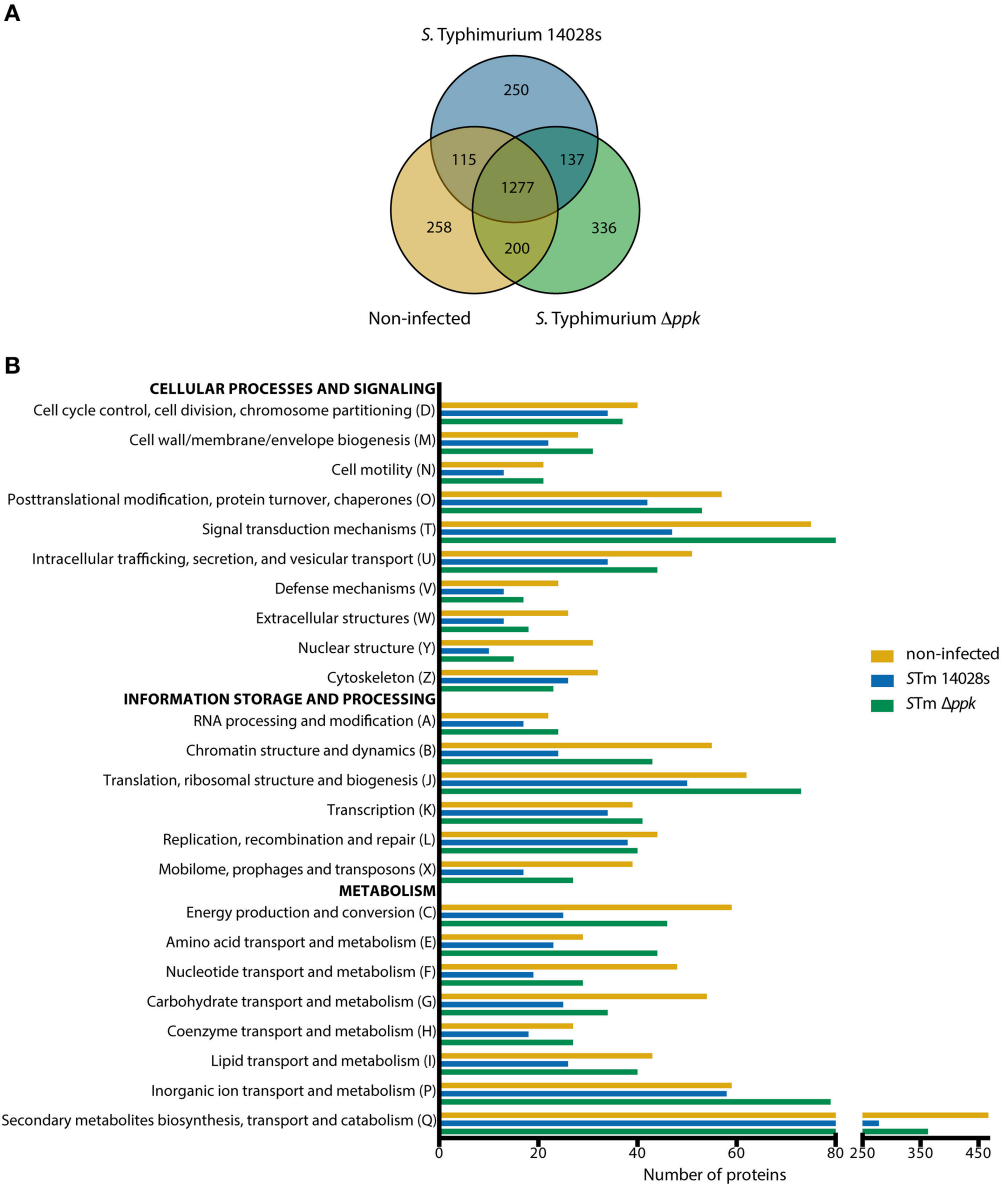
COG functional categorization of *D. discoideum* proteins detected during infection with *S*. Typhimurium strains. **(A)** Venn diagram of *D. discoideum* proteins detected in uninfected amoebae or in amoebae infected with *S*. Typhimurium wild-type or its Δ*ppk* derivative. **(B)** Graph showing number of proteins detected in each experimental condition and classified according to COG functional categories (see Materials and Methods section). COG categories were further grouped in three main classes: “Cellular processes and signaling”, “Information storage and processing”, and “Metabolism”.

A total of 258, 250, and 336 proteins were exclusively detected in non-infected amoebae, in amoebae infected with the wild-type strain, or in amoebae infected with the Δ*ppk* mutant, respectively. Additionally, 1277 proteins were detected in all three experimental conditions tested (Figure 3A). Considering that these proteins could be interesting if they show significant differences in expression levels, we carried out a comparative analysis of proteins detected in pairs of experimental conditions (i.e., non-infected amoebae vs. infected with the wild-type strain; non infected amoebae vs. infected with the Δ*ppk* mutant; and amoebae infected with the wild-type strain vs. infected with the Δ*ppk* mutant) to determine enrichment in a particular condition (cut-off: ~1.5 fold change). Proteins found exclusively or enriched in a given experimental condition were classified according to COG categories, which in turn were grouped in three main classes: “Cellular processes and signaling”, “Information storage and processing”, and “Metabolism” (Table S2). For most COG categories, the total number of proteins detected from non-infected amoebae was similar to those detected from amoebae infected with the Δ*ppk* mutant, in contrast to proteins detected in amoebae infected with the wild-type strain (Figure 3B). This was particularly evident in the case of COG categories “Posttranslational modification, protein turnover, chaperones”, “Signal transduction mechanisms”, “Intracellular trafficking, secretion, and vesicular transport” (associated with “Cellular processes and signaling”), “Chromatin structure and dynamics”, “Translation, ribosomal structure, and biogenesis”, “Mobilome, prophages, and transposons” (associated with “Information storage and processing”), “Energy production and conversion”, “Nucleotide transport and metabolism”, “Carbohydrate transport and metabolism”, “Coenzyme transport and metabolism”, “Lipid transport an metabolism”, and “Secondary metabolites biosynthesis, transport and catabolism” (associated with “Metabolism”; Figure 3B).

Additionally, proteins that were exclusive or significantly enriched in each experimental condition were identified and used to perform overrepresentation analyses using the PANTHER tool (Table S3). The results were filtered according to three annotation data sets: “Biological process”, “Cellular component”, and “Reactome pathways” (Table 3). Overrepresented groups of proteins detected in *D. discoideum* infected with either *S*. Typhimurium strain include those involved in endomembrane trafficking, actin cytoskeleton organization, social development, chemotaxis and response to cAMP, immune system, response to bacteria, ubiquitination and proteasome degradation.

**Table 3:** Overrepresentation analysis of *D. discoideum* proteins detected during infections with *S*. Typhimurium wild type and Δ*ppk*.

**A**. ***D. discoideum*** **infected with** ***S*****. Typhimurium wild type**		
**Biological process**	**Fold enrichment**	***p*****-value**
Actin filament reorganization (GO:0090527)	20.47	4.77E-02
Protein sulfation (GO:0006477)	20.47	4.77E-02
Positive regulation of protein localization to cell surface (GO:2000010)	20.47	4.77E-02
Anaerobic respiration (GO:0009061)	20.47	4.77E-02
Cell-cell signaling (GO:0007267)	20.47	4.77E-02
Rab protein signal transduction (GO:0032482)	20.47	4.77E-02
Positive regulation of single strand break repair (GO:1903518)	20.47	4.77E-02
Positive regulation of DNA repair (GO:0045739)	20.47	4.77E-02
Positive regulation of guanyl-nucleotide exchange factor activity (GO:1905099)	20.47	4.77E-02
Regulation of vacuole fusion, non-autophagic (GO:0032889)	20.47	4.77E-02
Lysosomal lumen acidification (GO:0007042)	20.47	4.77E-02
Secretion of lysosomal enzymes (GO:0033299)	12.28	2.02E-03
Protein localization to lysosome (GO:0061462)	10.24	1.68E-02
Negative regulation of phagocytosis (GO:0050765)	8.53	3.51E-04
Vesicle transport along microtubule (GO:0047496)	8.19	2.54E-02
Aerobic respiration (GO:0009060)	6.43	1.80E-06
DNA ligation involved in DNA repair (GO:0051103)	6.14	1.35E-02
Regulation of aggregate size involved in sorocarp development (GO:0031157)	5.69	2.10E-03
Response to reactive oxygen species (GO:0000302)	5.51	3.44E-04
Phosphatidylinositol phosphorylation (GO:0046854)	5.46	6.69E-03
Phosphatidylinositol-mediated signaling (GO:0048015)	4.82	1.02E-02
Response to cAMP (GO:0051591)	4.72	2.66E-02
Positive regulation of actin filament polymerization (GO:0030838)	4.09	3.93E-03
Defense response to bacterium (GO:0042742)	3.56	2.74E-02
Chemotaxis to cAMP (GO:0043327)	2.63	8.39E-03
Exocytosis (GO:0006887)	2.52	1.59E-02
Phagocytosis (GO:0006909)	2.44	9.34E-03
Aggregation involved in sorocarp development (GO:0031152)	1.87	2.46E-02
**Cellular component**	**Fold enrichment**	***p*****-value**
Proteasome core complex, beta-subunit complex (GO:0019774)	20.47	4.77E-02
Actomyosin, actin portion (GO:0042643)	20.47	4.77E-02
Site of double-strand break (GO:0035861)	20.47	4.77E-02
Nuclear SCF ubiquitin ligase complex (GO:0043224)	20.47	4.77E-02
Vacuolar proton-transporting V-type ATPase, V1 domain (GO:0000221)	13.65	9.73E-03
Early phagosome (GO:0032009)	7.31	7.00E-04
Proteasome core complex, alpha-subunit complex (GO:0019773)	5.85	4.66E-02
Phagolysosome (GO:0032010)	5.12	3.28E-03
Endosome (GO:0005768)	2.16	8.59E-03
**Reactome pathways**	**Fold enrichment**	***p*****-value**
G beta:gamma signaling through PI3Kgamma (R-DDI-392451)	20.47	4.77E-02
Antigen presentation: Folding, assembly and peptide loading of class I MHC (R-DDI-983170)	10.24	1.68E-02
Cell death signaling via NRAGE, NRIF and NADE (R-DDI-204998)	6.51	1.26E-04
Rho GTPases Activate WASPs and WAVEs (R-DDI-5663213)	6.40	1.26E-03
ROS, RNS production in response to bacteria (R-DDI-1222556)	6.14	1.35E-02
Iron uptake and transport (R-DDI-917937)	6.14	1.35E-02
PI3K Cascade (R-DDI-109704)	5.12	2.17E-02
Cytosolic sensors of pathogen-associated DNA (R-DDI-1834949)	4.39	3.21E-02
Cellular response to heat stress (R-DDI-3371556)	4.31	1.48E-02
Fc gamma receptor (FCGR) dependent phagocytosis (R-DDI-2029480)	4.24	3.34E-03
Signaling by Rho GTPases (R-DDI-194315)	3.32	3.64E-04
Cross-presentation of soluble exogenous antigens (endosomes) (R-DDI-1236978)	3.10	2.42E-02
Immune System (R-DDI-168256)	3.00	2.94E-07
Innate Immune System (R-DDI-168249)	2.58	4.63E-04
Adaptive Immune System (R-DDI-1280218)	2.53	2.39E-03
Antigen processing: Ubiquitination & Proteasome degradation (R-DDI-983168)	2.34	2.34E-02
Cellular responses to stress (R-DDI-2262752)	2.32	4.78E-02
**B. *D. discoideum* infected with *S*. Typhimurium Δ*ppk***		
**Biological process**	**Fold enrichment**	***p*****-value**
Positive regulation of sporulation (GO:0043938)	16.14	7.06E-03
Negative regulation of phagocytosis (GO:0050765)	8.07	1.23E-04
Arp2/3 complex-mediated actin nucleation (GO:0034314)	6.92	9.83E-03
Phosphatidylinositol 3-kinase signaling (GO:0014065)	6.46	3.91E-02
Regulation of exocytosis (GO:0017157)	6.46	3.91E-02
Secretion of lysosomal enzymes (GO:0033299)	6.46	3.91E-02
Regulation of protein ubiquitination (GO:0031396)	6.05	1.40E-02
Vacuolar acidification (GO:0007035)	6.05	1.40E-02
Negative regulation of actin filament polymerization (GO:0030837)	5.95	2.20E-04
Regulation of positive chemotaxis (GO:0050926)	5.38	1.91E-02
Response to cAMP (GO:0051591)	4.97	9.25E-03
Late endosome to vacuole transport (GO:0045324)	4.97	9.25E-03
Aerobic respiration (GO:0009060)	4.61	8.67E-05
Chemotaxis to cAMP (GO:0043327)	4.15	6.93E-07
Establishment of protein localization to vacuole (GO:0072666)	4.04	8.75E-03
Response to bacterium (GO:0009617)	3.99	3.18E-07
Actin filament polymerization (GO:0030041)	3.77	3.02E-03
Phagocytosis (GO:0006909)	3.65	2.21E-06
Response to oxidative stress (GO:0006979)	3.28	2.40E-04
Exocytosis (GO:0006887)	2.73	2.90E-03
Aggregation involved in sorocarp development (GO:0031152)	1.82	1.78E-02
**Cellular component**	**Fold enrichment**	***p*****-value**
Post-lysosomal vacuole (GO:0032195)	10.76	1.53E-02
Proton-transporting V-type ATPase, V1 domain (GO:0033180)	6.92	9.83E-03
Arp2/3 protein complex (GO:0005885)	6.46	3.74E-03
Early phagosome (GO:0032009)	5.76	1.98E-03
Proteasome complex (GO:0000502)	5.26	8.14E-07
Phosphatidylinositol 3-kinase complex (GO:0005942)	4.04	3.96E-02
Proteasome core complex (GO:0005839)	4.04	1.84E-02
Endosome (GO:0005768)	2.36	8.82E-04
Late endosome (GO:00057770)	2.36	8.82E-04
Lysosome (GO:0005764)	1.79	3.99E-02
**Reactome pathways**	**Fold enrichment**	***p*****-value**
eNOS activation and regulation (R-DDI-203765)	12.11	2.10E-03
Metabolism of nitric oxide (R-DDI-202131)	12.11	2.10E-03
ROS, RNS production in response to bacteria (R-DDI-1222556)	8.07	4.51E-04
Iron uptake and transport (R-DDI-917937)	6.73	1.02E-03
Cross-presentation of soluble exogenous antigens (endosomes) (R-DDI-1236978)	6.36	2.47E-07
Rho GTPases Activate WASPs and WAVEs (R-DDI-5663213)	5.04	3.50E-03
Fc gamma receptor (FCGR) dependent phagocytosis (R-DDI-2029480)	3.90	2.51E-03
Innate Immune System (R-DDI-168249)	3.35	5.65E-08
Signaling by Rho GTPases (R-DDI-194315)	3.05	2.87E-04
Antigen processing: Ubiquitination & Proteasome degradation (R-DDI-983168)	3.00	5.52E-04
Immune System (R-DDI-168256)	2.93	2.06E-08
Adaptive Immune System (R-DDI-1280218)	2.77	1.41E-04

*Overrepresentation analyses were performed using PANTHER (http://www.pantherdb.org/). UniProtKB IDs from exclusive and significantly-enriched proteins of D. discoideum detected during infections with the wild-type strain or the Δppk mutant were used as input. The results were filtered according to 3 annotation data sets: “Biological process,” “Cellular component,” and “Reactome pathways.” The cut-off for the analyses was set to p < 0.05*.

Regarding endomembrane trafficking, common overrepresented proteins include those involved in Secretion of lysosomal enzymes (GO:0033299), Exocytosis (GO:0006887), Phagocytosis (GO:0006909), Early phagosome (GO:0032009), Endosome (GO:0005768), Signaling by Rho GTPases (R-DDI-194315), Rho GTPases Activate WASPs and WAVEs (R-DDI-5663213), and Fc gamma receptor (FCGR) dependent phagocytosis (R-DDI-2029480). Overrepresented proteins in amoebae infected with the wild-type strain include those involved in Rab protein signal transduction (GO:0032482), Positive regulation of guanyl-nucleotide exchange factor activity (GO:1905099), Regulation of vacuole fusion, non-autophagic (GO:0032889), Lysosomal lumen acidification (GO:0007042), Protein localization to lysosome (GO:0061462), Vesicle transport along microtubule (GO:0047496), Phosphatidylinositol phosphorylation (GO:0046854), Phosphatidylinositol-mediated signaling (GO:0048015), G beta:gamma signaling through PI3Kgamma (R-DDI-392451), and PI3K Cascade (R-DDI-109704). On the other hand, overrepresented proteins in amoebae infected with the Δ*ppk* mutant include those linked to Phosphatidylinositol 3-kinase signaling (GO:0014065), Phosphatidylinositol 3-kinase complex (GO:0005942), Regulation of exocytosis (GO:0017157), Vacuolar acidification (GO:0007035), Late endosome to vacuole transport (GO:0045324), Establishment of protein localization to vacuole (GO:0072666), Post-lysosomal vacuole (GO:0032195), Late endosome (GO:00057770), and Lysosome (GO:0005764). It is well known that *S*. Typhimurium delivers T3SS-2 effector proteins that interfere with the maturation of the endocytic route in eukaryotic host cells in order to avoid phagolysosomal fusion. This process results in a unique vacuolar compartment referred to as the *Salmonella*-containing vacuole (SCV), where this pathogen resides (Haraga et al., 2008; LaRock et al., 2015). We have described that inactivation of T3SS-2 abolishes the intracellular survival of *S*. Typhimurium in *D. discoideum* (Riquelme et al., 2016). Accordingly, our analysis suggest that wild-type *S*. Typhimurium resides in an intracellular compartment of *D. discoideum* comparable to an early endosome, while the Δ*ppk* mutant resides in a compartment that ultimately fuses with the lysosome, explaining the defective intracellular survival phenotype shown by this strain in the amoeba (Figure 2).

Most overrepresented proteins associated with the response to bacterial infection were detected in amoeba infected with either strain of *S*. Typhimurium. These proteins include those linked to Immune System (R-DDI-168256), Innate Immune System (R-DDI-168249), Adaptive Immune System (R-DDI-1280218), Cross-presentation of soluble exogenous antigens (endosomes) (R-DDI-1236978), Antigen processing: Ubiquitination & Proteasome degradation (R-DDI-983168), and ROS, RNS production in response to bacteria (R-DDI-1222556). No overrepresented proteins were exclusively detected in infections with the wild-type strain, while overrepresented proteins only detected during infections with the Δ*ppk* mutant include those associated with Metabolism of nitric oxide (R-DDI-202131) and eNOS activation and regulation (R-DDI-203765). These proteins (SprA/Q54GP3, PtsA/Q1ZXI0, and GchA/Q94465) are required for the *de novo* biosynthesis of tetrahydrobiopterin, an essential co-factor for the aromatic amino acid hydroxylases and nitric oxide synthases in mammals (Thöny et al., 2000; Choi et al., 2006; Vásquez-Vivar, 2009). These results indicate that *D. discoideum* infected with either wild-type or Δ*ppk S*. Typhimurium generates a robust response that includes production of ROS and RNS in order to eliminate the pathogen.

Proteins exclusively detected in *D. discoideum* infected with the wild-type strain indicate that the pathogen induces DNA damage in the host. This is revealed by a number of proteins involved in Positive regulation of single strand break repair (GO:1903518), Positive regulation of DNA repair (GO:0045739), DNA ligation involved in DNA repair (GO:0051103), Site of double-strand break (GO:0035861), and Cell death signaling via NRAGE, NRIF, and NADE (R-DDI-204998), the latter being a process associated with apoptotic cell death. It is tempting to speculate that this DNA damage is the result of excessive ROS/RNS production in response to the pathogen. Furthermore, this DNA damage needs to be repaired by the amoeba in order to resume growth and development. This is in agreement with the ability of *S*. Typhimurium to delay the social development of *D. discoideum* according to our development assay (Figure 1). In addition, two proteins linked to Protein sulfation (GO:0006477) (i.e., Kil1/Q556K8 and Phg1a/Q55FP0) were only detected in amoeba infected with the wild-type strain. Both proteins have been implicated in *D. discoideum* killing of intracellular *K. pneumoniae* (Benghezal et al., 2006; Cosson and Soldati, 2008; Le Coadic et al., 2013). Of note, protein sulfation has been implicated in decreased bacterial adherence to eukaryotic cells and reduced T3SS-dependent cytotoxicity (Blondel et al., 2016). Thus, variations in sulfation levels on the surface of *D. discoideum* generated during *S*. Typhimurium infection may explain differences in internalization observed between the wild-type and attenuated strains Δ*ppk* and Δ*aroA* in our infection assays (Figure 2A).

Intracellular pathogens need to cope with a hostile environment inside the host during infection. Thus, our global proteomic profiles of *D. discoideum* proteins suggest that the amoeba recognizes wild-type *S*. Typhimurium and elicits a strong response that includes production of toxic ROS and RNS. Nevertheless, the pathogen manipulates the endocytic pathway of the host to generate an intracellular replicative niche to survive this cell-autonomous defense response. On the contrary, after recognition the Δ*ppk* mutant appears to be unable to subvert the amoebal autonomous defense mechanism and to survive the unfavorable conditions within the host. This idea is in agreement with the phenotypes reported for null mutants of *ppk* in several bacterial pathogens (Rao et al., 1998; Rashid and Kornberg, 2000; Rashid et al., 2000a,b; Kim et al., 2002; McMeechan et al., 2007; Brown and Kornberg, 2008; Varela et al., 2010; Gray and Jakob, 2015; Varas et al., 2017). In addition the mutant seems to be incapable to modify the endocytic pathway, ending in a degradative intracellular compartment. Consequently, the Δ*ppk* mutant is unable to survive within the amoeba and to subvert the social development of this host.

**Figure 4:**
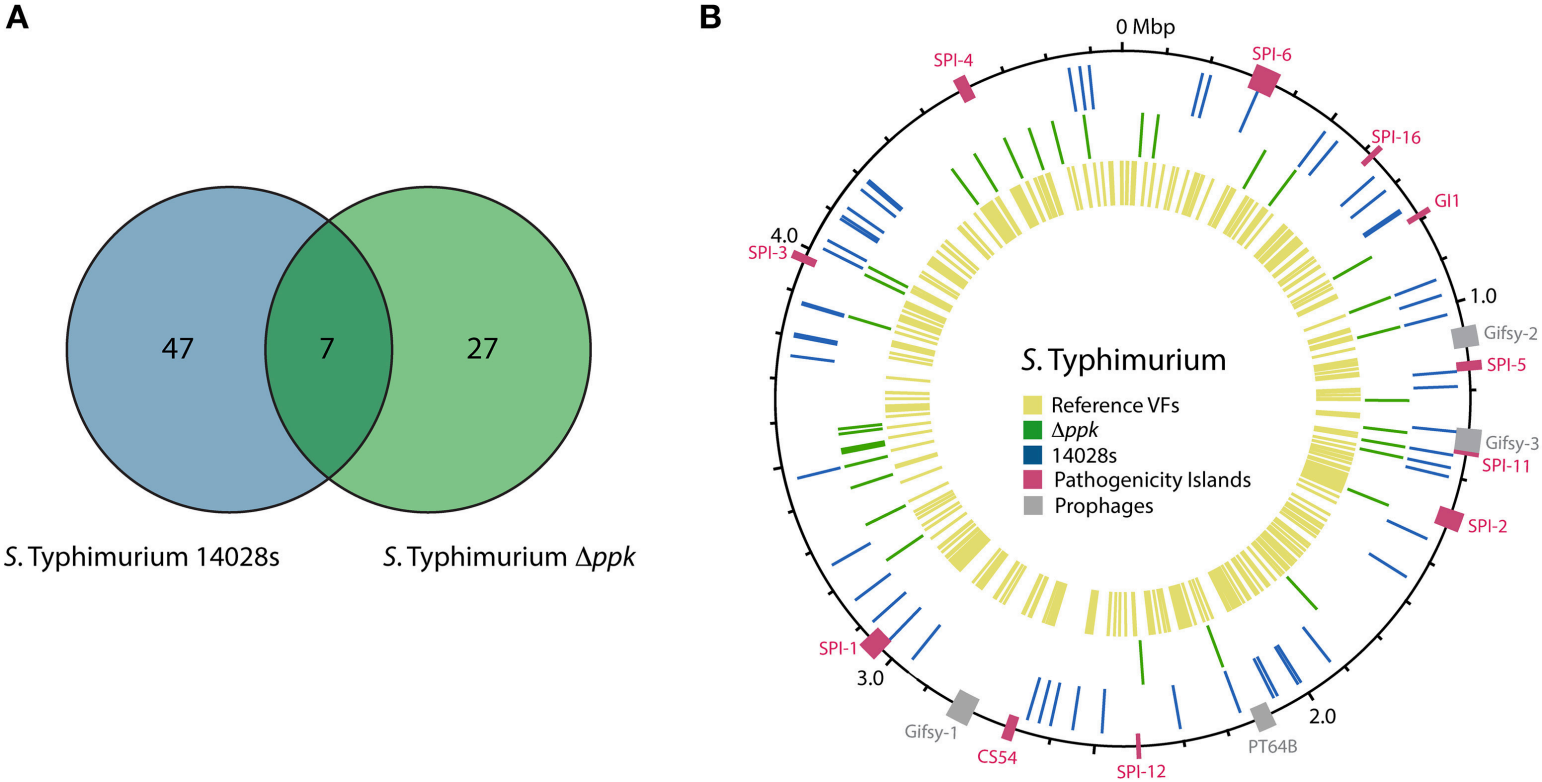
*S*. Typhimurium proteins detected by global proteomic profiling during infection of *D. discoideum*. **(A)** Venn diagram of *S*. Typhimurium proteins detected during infection of *D. discoideum* with the wild-type strain or its Δ*ppk* derivative. **(B)** Circular representation of *S*. Typhimurium 14028s chromosome. Boxes in magenta and gray indicate regions corresponding to known *Salmonella* pathogenicity islands (SPIs) and prophages, respectively. Blue and green lines indicate the location of genes encoding proteins detected during the infection with the wild-type strain or its Δ*ppk* derivative, respectively. Yellow lines indicate the location of genes encoding virulence-associated proteins listed in databases PATRIC_VF, VFDB, and VICTORS (see Materials and Methods section).

In addition to *D. discoideum* proteins, we attempted to identify *S*. Typhimurium proteins expressed during the infection of the amoeba with the wild-type strain or the Δ*ppk* mutant. Using our Q-proteomics approach we were able to detect a limited number of bacterial proteins, most probably due to their low relative abundance in each sample in comparison to *D. discoideum* proteins. Thus, a total of 54 and 34 proteins were identified in infections of amoeba cells with the wild-type strain and the Δ*ppk* mutant, respectively. A group of seven proteins was detected in amoebae infected with either strain (Figure 4A). The identities of all *S*. Typhimurium proteins detected are listed in Table 4. The location of genes encoding all these proteins in the genome of *S*. Typhimurium 14028s is shown in Figure 4B. The list of detected proteins was compared with a list of 469 classic virulence-related proteins included in databases PATRIC_VF, VFDB and VICTORS using tools implemented in PATRIC (www.patricbrc.org). We identified 11 *S*. Typhimurium proteins linked to virulence in amoebae infected with the wild-type strain, and 4 of these proteins in amoebae infected with the Δ*ppk* mutant, respectively (proteins highlighted in bold type in Table 4).

**Table 4:** *S*. Typhimurium proteins detected during infections of *D. discoideum* with the wild-type strain or the Δ*ppk* mutant.

**Locus tag in strain 14028s**	**Locus tag in strain LT2**	**Gene name**	**Product**
**A. Infection with the wild-type strain**			
STM14_0190	STM0158	*acnB*	Bifunctional aconitate hydratase 2/2-methylisocitrate dehydratase
STM14_0207	STM0175	*stiC*	Putative fimbrial usher
STM14_0329	STM0281	–	Putative cytoplasmic protein
**STM14_0529**	**STM0447**	***tig***	**Trigger factor**
STM14_0564	STM0479	–	Putative transposase
STM14_0693	STM0595	*entC*	Isochorismate synthase
STM14_0743	STM0636	*ybeD*	Hypothetical protein
STM14_0806	STM0691	–	Tricarballylate dehydrogenase
STM14_0815	STM0698	*pgm*	Phosphoglucomutase
**STM14_1013**	**STM0863**	***dacC***	**D-alanyl-D-alanine carboxypeptidase fraction C**
STM14_1056	STM0939	*ybjD*	Hypothetical protein
**STM14_1106**	**STM0978**	***aroA***	**3-phosphoshikimate 1-carboxyvinyltransferase**
STM14_1259	STM1109	–	Putative periplasmic protein
STM14_1309	STM1143	*csgB*	Curlin minor subunit
STM14_1432	–	–	Phage replication protein O
**STM14_1515**	**STM1255**	–	**Putative ABC transporter periplasmic binding protein**
**STM14_1565**	**STM1290**	***gapA***	**Glyceraldehyde-3-phosphate dehydrogenase**
STM14_1590	STM1310	*nadE*	NAD synthetase
STM14_1770	STM1468	*fumA*	Fumarase A
STM14_1884	STM1561	–	Putative lipoprotein
STM14_2183	STM1806	*nhaB*	Sodium/proton antiporter
STM14_2303	STM1894	*ruvB*	Holliday junction DNA helicase B
STM14_2313	–	–	Hypothetical protein
STM14_2383	STM1963	*amyA*	Cytoplasmic alpha-amylase
STM14_2395	STM1974	*fliK*	Flagellar hook-length control protein
STM14_2515	STM2027	*cbiH*	Precorrin-3B C17-methyltransferase
STM14_2658	STM2155	*metG*	Methionyl-tRNA synthetase
STM14_2864	STM2323	*nuoG*	NADH dehydrogenase subunit G
STM14_2938	STM2389	*fadI*	3-ketoacyl-CoA thiolase
STM14_2999	STM2441	*cysA*	Sulfate/thiosulfate transporter subunit
STM14_3031	STM2472	*maeB*	Malic enzyme
STM14_3058	–	–	Hypothetical protein
STM14_3368	STM2792	*gabT*	4-aminobutyrate aminotransferase
STM14_3463	STM2866	*sprB*	Transcriptional regulator
STM14_3529	STM2926	*pcm*	Protein-L-isoaspartate O-methyltransferase
STM14_3594	STM2980	*ygdE*	Putative RNA 2'-O-ribose methyltransferase
STM14_3709	STM3069	*pgk*	Phosphoglycerate kinase
**STM14_3969**	**STM3286**	***infB***	**Translation initiation factor IF-2**
STM14_4299	STM3571	*ftsY*	Cell division protein FtsY
STM14_4345	STM3610	*yhjG*	Putative inner membrane protein
STM14_4351	STM3614	*dctA*	C4-dicarboxylate transporter DctA
STM14_4431	STM3674	*lyxK*	L-xylulose kinase
**STM14_4435**	**STM3678**	–	**Putative regulatory protein (AraC family)**
STM14_4585	STM3796A	–	Integral membrane protein
STM14_4614	STM3822	*torA*	Trimethylamine N-oxide reductase subunit
STM14_4665	STM3869	*atpF*	F0F1 ATP synthase subunit B
STM14_4675	STM3878	*yieM*	Hypothetical protein
STM14_4698	STM3901	*ilvG*	Acetolactate synthase 2 catalytic subunit
STM14_4760	STM3957	*pldA*	Phospholipase A
STM14_4785	STM3738	*yigC*	3-octaprenyl-4-hydroxybenzoate decarboxylase
STM14_4789	STM3983	*fadB*	Multifunctional fatty acid oxidation complex subunit alpha
STM14_5386	STM4489	–	Putative DNA helicase
STM14_5404	STM4503	–	Putative inner membrane protein
STM14_5437	STM4525	*hsdM*	DNA methylase M
**B. Infection with the Δ*ppk* mutant**			
STM14_0055	STM0046	*ileS*	Isoleucyl-tRNA synthetase
STM14_0100	STM0084	–	Putative sulfatase
STM14_0417	STM0357	*mod*	DNA methylase
**STM14_0529**	**STM0447**	***tig***	**Trigger factor**
STM14_0870	STM0748	*tolB*	Translocation protein TolB
STM14_0992	STM0798	*uvrB*	Excinuclease ABC subunit B
**STM14_1013**	**STM0863**	***dacC***	**D-alanyl-D-alanine carboxypeptidase fraction C**
**STM14_1106**	**STM0978**	***aroA***	**3-phosphoshikimate 1-carboxyvinyltransferase**
STM14_1353	STM1182	*flgJ*	Peptidoglycan hydrolase
STM14_1459/STM14_3188	STM2605	–	Prophage head-tail preconnector
**STM14_1515**	**STM1255**	–	**Putative ABC transporter periplasmic binding protein**
**STM14_1565**	**STM1290**	***gapA***	**Glyceraldehyde-3-phosphate dehydrogenase**
STM14_1723	STM1426	*ribE*	Riboflavin synthase subunit alpha
STM14_2116	STM1751	*hns*	Global DNA-binding transcriptional dual regulator H-NS
STM14_2516	STM2028	*cbiG*	Cobalamin biosynthesis protein CbiG
STM14_2753	STM2227	*yejL*	Hypothetical protein
STM14_3634	STM3010	*aas*	Bifunctional acyl-[acyl carrier protein] synthetase/2-acylglycerophosphoethanolamine acyltransferase
STM14_3771	STM3122	–	Putative arylsulfatase
STM14_3899	STM3220	*ygjO*	Putative methyltransferase
**STM14_3969**	**STM3286**	***infB***	**Translation initiation factor IF-2**
STM14_4016	STM3328	*arcB*	Aerobic respiration control sensor protein ArcB
STM14_4020	STM3330	*gltB*	Glutamate synthase subunit alpha
STM14_4022	STM3332	*yhcG*	Putative cytoplasmic protein
STM14_4067	STM3373	*mreC*	Cell wall structural complex MreBCD transmembrane component MreC
STM14_4083	STM3385	*fis*	DNA-binding protein Fis
**STM14_4435**	**STM3678**	–	**Putative regulatory protein (AraC family)**
STM14_4568	STM3787	*uhpT*	Sugar phosphate antiporter
STM14_4599	STM3808	*ibpB*	Heat shock chaperone IbpB
STM14_4982	STM4146	*tuf_2*	Elongation factor Tu
STM14_5066	STM4213	–	Putative phage tail sheath protein
STM14_5155	STM4285	*fdhF*	Formate dehydrogenase
STM14_5237	STM4356	*yjeF*	Hypothetical protein
STM14_5312	STM4421	–	Putative NAD-dependent aldehyde dehydrogenase
STM14_5395	STM4498	–	Putative inner membrane protein

*Bold type indicates proteins detected during infection with either the wild-type strain or the Δppk mutant. Red indicates classic virulence-related proteins included in databases PATRIC_VF, VFDB, and VICTORS that were identified using tools implemented in PATRIC (www.patricbrc.org)*.

The limited amount of *S*. Typhimurium proteins detected in our global proteomic profiling impeded us conducting an insightful comparative analysis in order to understand differences between infections of *D. discoideum* with the wild-type strain and the Δ*ppk* mutant. However, it is worth mentioning a particular group of *S*. Typhimurium proteins detected during amoebae infections that includes AroA, SprB, STM14_0329, H-NS, Fis, ArcB, NuoG, EntC, IbpB, CsgB, StiC, and FliK.

AroA is a 3-enolpyruvylshikimate-5-phosphate synthetase that is crucial for biosynthesis of aromatic compounds. As a result, *Salmonella aroA* null-mutants are highly attenuated in different infection models (Hoiseth and Stocker, 1981; Stocker et al., 1983; Cooper et al., 1990) and present strong defects in survival within macrophages (Fields et al., 1986; Lowe et al., 1999) and in *D. discoideum* (Riquelme et al., 2016). This protein was detected in amoebae infected with both *S*. Typhimurium wild-type and Δ*ppk* strains.

SprB is a transcription factor from the LuxR/UhaP family that is encoded in SPI-1. This protein regulates the coordinate expression of SPI-1 and SPI-4 genes during *Salmonella* infection (Saini and Rao, 2010). STM14_0329 (also known as SciO and TssK) is one of the 13 core components of the T6SS encoded in SPI-6 (Blondel et al., 2009; Journet and Cascales, 2016). T6SS are versatile weapons exploited by numerous bacterial pathogens to target either eukaryotic host cells or competitor bacteria (Cianfanelli et al., 2016; Hachani et al., 2016; Journet and Cascales, 2016). Noteworthy, we have recently described that null mutations in *S*. Typhimurium genes encoding essential components of T3SS-1 and SPI-6 T6SS cause intracellular survival defects in *D. discoideum* (Riquelme et al., 2016).

The nucleoid-associated protein H-NS selectively silences horizontally-acquired genes by direct binding to DNA sequences with high TA content in the genome, including all major SPIs in *S*. Typhimurium (Lucchini et al., 2006; Navarre et al., 2006). Accordingly, *hns* mutations are highly pleiotropic in *S*. Typhimurium (Hinton et al., 1992) and produce attenuated strains (Harrison et al., 1994). A recent study indicates that the fitness defects presented by *hns* mutants of *S*. Typhimurium are mainly due to a misregulation of SPI-1 genes (Ali et al., 2014). The factor for inversion stimulation (Fis) is a nucleoid-associated protein that influences the topological state of DNA in the cell by direct binding to DNA and by modulating DNA gyrase and topoisomerase I gene expression. As in the case of H-NS, Fis acts as a key regulator of virulence in *S*. Typhimurium mainly by controlling the coordinate expression of genes located in several SPIs, as well as genes involved in motility (reviewed in Duprey et al., 2014). Therefore, *fis* mutants of *S*. Typhimurium are defective for intracellular survival in macrophages (O Cróinín et al., 2006; Wang et al., 2013). ArcB is the sensor component of the master regulatory two-component system ArcA/ArcB, that controls the expression of several genes and operons encoding proteins linked to the metabolic shift from anaerobic to aerobic conditions, and the enzymatic defenses of bacteria against ROS (Evans et al., 2011). It is worth mentioning that H-NS, Fis and ArcB were only detected in amoebae infected with the Δ*ppk* mutant, perhaps reflecting adjustments required to cope with the pleiotropic phenotypes presented by this kind of mutant.

NuoG is a subunit of the NADH dehydrogenase I complex. *S*. Gallinarum Δ*nuoG* mutants are attenuated in chicken and show reduced survival and multiplication in the reticuloendothelial system of this host (Zhang-Barber et al., 1998; Turner et al., 2003). Noteworthy, it has been reported that NuoG is involved in detoxification of ROS produced by macrophages during *M. tuberculosis* infection (Miller et al., 2010). Thus, it is tempting to speculate that NuoG can play a similar role during *S*. Typhimurium infection of *D. discoideum*.

EntC is an isochorismate synthase involved in the biosynthesis of catecholate siderophores enterobactin and salmochelin, produced by *Salmonella* (and other bacterial pathogens) to capture iron from the host during infection (reviewed in Fischbach et al., 2006). It has been established that production of salmochelin is essential for full virulence of *S*. Typhimurium in mice (Crouch et al., 2008). Furthermore, the production of enterobactin and salmochelin is required for *S*. Typhimurium to survive in macrophages at early stages of infection, and these siderophores protect the pathogen against reactive oxygen species produced by macrophages during the infective process (Achard et al., 2013). Our results suggest that *S*. Typhimurium produces catecholate siderophores inside *D. discoideum*, perhaps contributing to the intracellular survival of this pathogen.

IbpB is a small heat-shock protein (sHSP) being member of a widely conserved family of ATP-independent molecular chaperones that bind to misfolded proteins and protect them from irreversible aggregation (Laskowska et al., 1996; Lee et al., 1997). It has been reported that *E. coli* chaperones IbpB and IbpA are substrates for the ATP-dependent Lon protease (Bissonnette et al., 2010). In addition, polyP forms a complex with Lon and stimulates the degradation of selected proteins (Kuroda et al., 2001, 2006; Nomura et al., 2004; Kuroda, 2006). Thus, there is a functional link between IbpB, Lon and the biosynthesis of polyP during the bacterial stress response.

Among the bacterial proteins detected in amoebae infected with wild-type *S*. Typhimurium we found CsgB, StiC, and FliK, which are associated to the assembly of proteinaceous structures such as fimbriae and flagellum, respectively. This is noteworthy because it is generally accepted that this kind of surface structures are repressed upon invasion of host cells. CsgB participates in the assembly of the curli fimbriae, favoring the polymerization of its major component CsgA (reviewed in Evans and Chapman, 2014). Curli fimbriae are amyloid fibers that act as scaffolding agents in biofilms of *E. coli* and *Salmonella*, providing increased resistance to desiccation and to sodium hypochlorite, and being extremely resistant to proteolysis and chemical denaturation (Chapman et al., 2002; White et al., 2006). These fimbriae have been linked to cell-cell contacts, and to adherence to various eukaryotic cells, tissues, and abiotic surfaces, thus promoting community behavior and host colonization, playing an important role in the initial stages of the infection process (Barnhart and Chapman, 2006). To our knowledge, there are no previous reports on the expression of CsgB when *Salmonella* resides inside host cells. StiC is an usher protein encoded in one of the 11 chaperone-usher fimbrial gene clusters in the genome of *S*. Typhimurium (Jarvik et al., 2010). Chaperone-usher fimbriae normally have one or more structural subunits, which are exported and assembled on the bacterial surface by cognate periplasmic chaperone proteins and an outer-membrane usher protein. Some of them have demonstrated roles in binding to different receptors, persisting in specific niches, promoting infections, or forming biofilms (Weening et al., 2005; Clayton et al., 2008; Yue et al., 2012). The *stiABCH* gene cluster encodes a class γ-fimbriae, characterized for harboring subunits comprising the domains PFAM00419 or COG3539 (Nuccio and Bäumler, 2007). To our knowledge, no previous studies have addressed the specific contribution of this fimbrial operon to *S*. Typhimurium virulence. However, a previous study showed that the chaperone-usher SEF14 fimbriae are essential for an efficient uptake and survival of *S*. Enteritidis in murine macrophages, suggesting that they may be required at stages beyond the initial host colonization (Edwards et al., 2000). FliK is the protein that controls the length of the flagellar hook, and is encoded in one of the 17 operons composing the flagellar regulon of *S*. Typhimurium (Chilcott and Hughes, 2000). The flagellum is required for bacterial access to the intestinal epithelium, adherence to several tissues, and immune modulation (Rossez et al., 2015). Although flagellum assembly is normally prevented inside host cells, it was previously reported that intracellular *Salmonella* triggers swelling of macrophages (referred to as “oncotic macrophages”) in a process where flagellated bacilli intermittently escape from infected host cells (Sano et al., 2007).

## Conclusions

Overall, our results indicate that polyP biosynthesis is crucial for virulence and intracellular survival of *S*. Typhimurium in *D. discoideum*. In addition, we have validated the use of global proteomic analyses to gain insight into the host-pathogen interaction of *D. discoideum* and *S*. Typhimurium. The analysis of host proteins related to endocytic pathway, immune response, cell death, cytoskeleton dynamics, and developmental process revealed mechanisms that may explain the phenotypes shown by a *S*. Typhimurium strain lacking polyP during *D. discoideum* infection. Thus, our work demonstrates that unbiased high-throughput proteomics can be used as a powerful approach to provide new perspectives on host-pathogen interactions. Furthermore, our infection and development assays using these organisms can be exploited to screen for novel anti-virulence molecules targeting inorganic polyP biosynthesis.

## Author contributions

MV, SR-B, CV, CS, and FC: Conceived and designed the experiments; MV, SR-B, CV, and AM: Performed the experiments; MV, SR-B, CV, AM, CB-P, CS, and FC: Analyzed the data; MV, CB-P, CS, and FC: Contributed with reagents/animals/materials/analysis tools; MV, AM, CS, and FC: Wrote the paper. All authors read and approved the final manuscript.

### Conflict of interest statement

The authors declare that the research was conducted in the absence of any commercial or financial relationships that could be construed as a potential conflict of interest.

## Supplementary material

Table S1 List of all proteins detected in uninfected amoebae, and in amoebae infected with *S*. Typhimurium wild type or Δ*ppk*, and their corresponding PANTHER annotation.

Table S2 Proteins found exclusively or enriched in a given experimental condition, classified according to COG categories.

Table S3 Overrepresentation analysis for proteins found exclusively or enriched in amoebae infected with *S*. Typhimurium wild type or Δ*ppk*.
